# Urological Examination Compared to Ultrasonography in Testicular Lump Assessment: A Retrospective Cohort Study

**DOI:** 10.7759/cureus.72346

**Published:** 2024-10-25

**Authors:** Rahel Rashid, Baidar Khalabazyane, Charlotte Bee, Mohamed Ali, Thomas Pugh, Luke Hanna, Israa Kadhmawi, Roza Salah, Joshua Philips

**Affiliations:** 1 General and Colorectal Surgery, Arrowe Park Hospital, Wirral, GBR; 2 Urology, Royal Bournemouth Hospital, Bournemouth , GBR; 3 Urology, Royal Bournemouth Hospital, Bournemouth, GBR; 4 Surgery, Arrowe Park Hospital, Wirral, GBR; 5 Plastic and Reconstructive Surgery, Salisbury Foundation Trust, Bournemouth, GBR

**Keywords:** epididymo-orchitis, hydrocele, scrotal lump, scrotal ultrasound, testicular cancer

## Abstract

Background

General practitioners (GPs) often expedite indeterminate scrotal lumps for urological evaluation. While a scrotal examination by a urologist is crucial, ultrasound (US) has become a routine component of clinical assessment regardless of the clinical examination findings and the nature of the symptoms. This study aimed to evaluate the efficacy of clinical examination compared to scrotal ultrasound, even when the suspicion of cancer was low.

Methodology

A retrospective review of all fast-track testicular referrals seen in the clinic between January 2018 and January 2021 was conducted. Data on clinical examination findings, ultrasound results, and final diagnoses were analyzed. Patients for whom ultrasound scans were available before clinical examination were excluded from the study to avoid confounding the results.

Results

A total of 398 male subjects were referred for urological assessment, and 123 cases were excluded based on specified exclusion criteria. Two hundred seventy-five patients were identified who underwent clinical examination by urologists and subsequent ultrasound scans. Among 30 (11%) potentially malignant cases, 18 (60%) were confirmed malignancies. Sixty-eight (24.7%) cases were deemed *unlikely to be malignant*, and an ultrasound scan confirmed 40 (58.8%) cases as normal and four (5.9%) cases of unexpected malignancy. Ultrasonography confirmed 19 of 27 hydroceles (70.4%), 64 of 89 epididymal cysts (71.9%), and 5 of 9 varicoceles (55.6%). Of 51 epididymo-orchitis cases, 14 (27.5%) were confirmed.

Conclusions

Urological examinations demonstrated high reliability in most cases, with clinical diagnoses frequently corroborated by ultrasonographic findings. The results indicate that when there is no clinical indication for an ultrasound scan, it is more efficient to avoid unnecessary ultrasonography, as it can be time-consuming without providing additional diagnostic benefits. This underscores the value of thorough clinical assessment in guiding the need for further imaging.

## Introduction

Scrotal lumps or swelling are fairly common and may arise from trauma, infection, benign conditions such as cysts or hydrocele, or malignant pathologies. They can be detected through self-examination or by a partner. Typically, in the United Kingdom, a patient's first step is to consult their General Practitioner (GP). To rule out any serious conditions, these patients often undergo an ultrasound (US) scan in the community and/or are referred to a specialist urological service for further evaluation. Despite scrotal lumps’ prevalence, testicular cancer is a relatively uncommon finding, with an annual incidence of 3-10/100,000 men [1].

Ultrasonography is an ideal, non-invasive imaging technique for assessing scrotal abnormalities, particularly in differentiating key causes of acute scrotal pain and swelling. It is also the primary imaging method for evaluating acute scrotal trauma as it can detect conditions such as tunica albuginea rupture, hematomas, and contusions [2]. In cases of palpable abnormalities or scrotal swelling, ultrasonography effectively detects, locates, and characterizes both intra-testicular and extra-testicular masses, while also providing valuable insight into vascular perfusion through color and spectral Doppler analysis. Typically, a combination of clinical history, physical examination, and ultrasonographic findings is sufficient for accurate diagnosis [3].

Ultrasonography not only identifies and characterizes lesions but can often differentiate between benign and malignant conditions, highlighting those requiring urgent surgical intervention. Moreover, ultrasonography is a safe, cost-effective, and widely accessible modality that avoids the use of ionizing radiation. However, a US scan is a particularly operator-dependent investigation, which can sometimes limit its potential.

Nonetheless, as the most common cause for scrotal lumps is benign (extra-testicular) lesions [4], and US is the first diagnostic tool available, this can result in unnecessary requests and can sometimes become a reassuring or confirmatory tool without offering much diagnostic value [5].

Limited research has compared the accuracy of clinical examinations compared to US in detecting clinically significant lesions. In most studies, lesions identified solely by US, if clinically insignificant, did not influence patient management [6]. Additionally, scrotal US holds limited diagnostic value in patients with chronic scrotal pain when both physical examination and urinalysis are normal [7].

This study aims to compare urologists' clinical examinations with ultrasonography in the evaluation of scrotal lumps and swelling.

## Materials and methods

Study design and participants

This study employed a retrospective review of medical records from fast-track testicular referrals seen at our clinic from January 2018 to December 2020. We included all men aged 18 years and above who were referred for the evaluation of scrotal lumps or testicular pain and swelling that persisted for 14 days or more. To minimize potential confounders and biases, we applied specific exclusion criteria. These included cases that had received US scans or vetting before specialist urological review, as well as cases that were discharged without requesting any US scan. Additionally, patients presenting with acute scrotal pain or swelling (less than 14 days duration) were not included in the study cohort.

Data collection and analysis

Data collection involved extracting information from electronic patient records (EPRs), encompassing patient demographics, clinical examination findings, US results, and final diagnoses. Our primary outcome measure was the concordance between clinical examination findings and US results. We placed particular emphasis on identifying cases where US revealed unexpected malignancy or other significant findings that were not suspected during the clinical examination. This approach allowed us to assess the effectiveness of clinical examinations in detecting testicular abnormalities and evaluate the added diagnostic value of US scans in this context.

**Figure 1:**
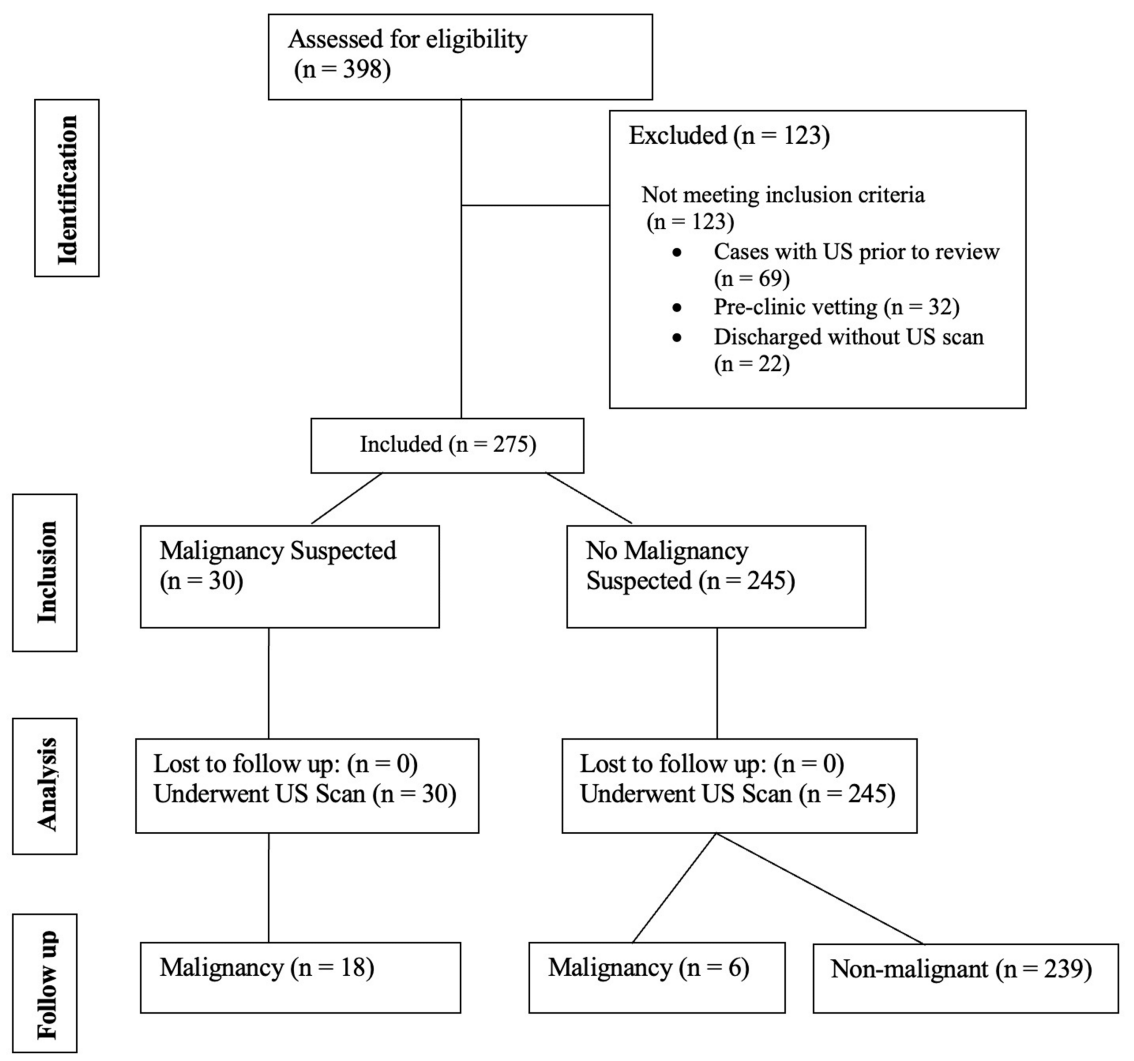
STROBE diagram showing patients' flow. n, number; US, ultrasound; STROBE, Strengthening the Reporting of Observational Studies in Epidemiology

The study design adhered to the Strengthening the Reporting of Observational Studies in Epidemiology (STROBE) guidelines, ensuring a standardized and comprehensive approach to reporting our observational research (Figure 1).

## Results

During the study period, 398 male patients were referred for expedited testicular assessment. The median age was 28 years (range 18 to 73). After applying the exclusion criteria: within the cohort, 69 (17.3%) subjects had undergone prior ultrasonographic scan (USS) requested by primary care providers, pre-clinic vetting of ultrasonographic results had been performed for 32 (8%) patients, and following clinical evaluation, 22 (5.5%) individuals were discharged without requesting further ultrasonographic investigation. This resulted in a study sample of 275 subjects who underwent ultrasonography following clinical assessment by urologists. 

Based on clinical examination, we found 89 (32.3%) epididymal cysts, 30 (11%) suspected malignancies, and 51 (18.5%) cases of epididymo-orchitis. Additionally, 9 (3.3%) cases of varicocele, 27 (10%) cases of hydrocele, and one case (0.36%) of hematoma were identified. Notably, 68 (24.7) cases were classified as normal non-malignant, with no significant abnormalities on examination.

In the 30 cases initially classified as potentially malignant, ultrasonographic findings confirmed malignancy in 18 instances (60%). Additionally, ultrasonography identified two cases (7%) as epididymal cysts, four cases (13%) as epididymo-orchitis, and six cases (20%) with normal scans.

Among the 68 cases labeled as *unlikely malignant*, ultrasonographic results revealed 41 normal scans (60.3%), four malignancies (5.9%), 18 cysts (26.5%), two epididymo-orchitis (2.9%), one hydrocele (1.5%), and two varicoceles (2.9%).

Of the 27 cases initially diagnosed as hydrocele, ultrasonographic evaluation confirmed 19 cases (70.4%) as hydrocele, four cases (14.8%) as normal, two cases (7.4%) as malignancies, and two cases (7.4%) as epididymal cysts.

For the 89 cases initially identified as epididymal cysts, ultrasonography confirmed 64 cases (71.9%), 20 cases (22.5%) as normal, and five cases (5.6%) as hydrocele.

Among the nine cases initially diagnosed as varicocele, ultrasonographic evaluation confirmed five cases (55.6%), three cases (33.3%) as normal, and one case (11.1%) as an epididymal cyst.

Of the 51 instances identified as epididymo-orchitis, ultrasonographic evaluation confirmed 14 cases (27.5%), 23 cases (45%) as normal, 10 cases (19.6%) as epididymal cysts, and four cases (7.8%) as varicocele. In the single case initially labeled as a hematoma, it was corroborated by USS. Table 1 summarizes the results.

**Table 1:** Summary of clinical and ultrasound findings. N, number; N/A, not applicable; PPV, positive predictive value

Clinical examination (*N*)	Ultrasound confirms (%) (PPV)	Ultrasound disagrees
Potentially malignant (30)	18 (60)	2 epididymal cyst
		4 epididymo-orchitis
		6 normal
“Unlikely malignant” (68)	41 (60.3) normal	4 malignancy
	18 (26.5) epididymal cyst	
	2 (2.9) epididymo-orchitis	
	2 (2.9) varicocele	
	1 (1.5) hydrocele	
Hydrocele (27)	19 (70.4)	4 normal
		2 malignancy
		2 epididymal cyst
Epididymal cyst (89)	64 (71.9)	20 normal
		5 hydrocele
Varicocele (9)	5 (55.6)	3 normal
		1 epididymal cyst
Epididymo-orchitis (51)	14 (27.5)	23 normal
		10 epididymal cyst
		4 varicocele
Hematoma (1)	1 (100%)	N/A
Total (275)	185 (67%)	90 (33%)

## Discussion

The US uses a high-frequency transducer and color Doppler analysis is the primary imaging modality for assessing scrotal lesions. When evaluating a palpable mass with the US, the main objectives are to determine the mass's location (intra-testicular or extra-testicular) and to further characterize it as either solid or cystic. An intra-testicular solid mass is highly suspicious for malignancy, with over 95% of such lesions being malignant. In contrast, extra-testicular lesions, which are more common, are usually benign [4,8,9]. This has recently been reviewed by Gabriel et al., who agreed that while most extra-testicular lesions are indeed benign, this is largely due to the prevalence of conditions like spermatoceles, lipomas, or epididymal cysts. If these benign conditions are excluded from consideration, the incidence of malignancy among the remaining lesions, specifically if solid, significantly increases [10]. 

Our study cohort was 275 patients who were referred to the specialist urological service via a fast-track pathway. This is a dedicated pathway in the United Kingdom for cases of suspected malignancy. Which is to say, these patients were referred to diagnose or rule out testicular cancer. The literature shows that the majority of testicular pathologies can be diagnosed based on physical examination alone [11] and that the scrotal US should be reserved for specific circumstances. 

From 275 cases of fast-track cases that underwent subsequent specialist urological examination, only 30 cases (11%) remained suspicious for malignancy. From this cohort, 18 (60%) cases were confirmed to have malignancy in the US. The remaining 245 cases (89%) were not suspected to be testicular cancer. While this was the case in 239 (97.5%) cases, six cases (2.4%) were diagnosed as malignancy. 

This highlights the significant reliability of urological examination in diagnosing testicular conditions, with a high concordance between clinical assessments and ultrasonographic results in most cases, this is also reflected in the study by van Haarst et al., in which they emphasize the importance of thorough physical examination and doctors regaining confidence in their ability to perform this [7]. 

The results demonstrate that clinical examinations were particularly accurate in identifying epididymal cysts, hydroceles, and varicoceles, with ultrasonography confirming 71.9%, 70.4%, and 55.6% of these diagnoses, respectively. If we adjust these for non-malignant findings, the sensitivity will be even higher. This high level of accuracy suggests that experienced urologists can often make correct diagnoses through careful physical examination alone, and reassure clinicians that unpalpable testicular neoplasms are extremely uncommon [12].

However, this is not to suggest that ultrasonography is not required for cases that present with a scrotal lump where malignancy is not suspected. On the contrary, any case that presents with a scrotal lump above 14 days should be investigated using a US scan [6], as our data showed the finding of six (out of 245) cases (2.44%) of malignancy among the cohort for which malignancy was not suspected. This is supported within the literature among several aspects; first, the predictive value of a positive clinical examination can be low at times [13]. Second, some testicular tumors have been discovered incidentally on US imaging, despite the absence of a palpable scrotal lump [14]. Third, testicular malignancies can sometimes be misdiagnosed as other clinical conditions, the most common being epididymitis [15,16]. 

The study also revealed that ultrasonography was less effective in confirming cases of epididymo-orchitis, with only 27.5% of clinically diagnosed cases confirmed by imaging. This discrepancy might be attributed to the dynamic nature of inflammatory conditions, which can change rapidly between clinical examination and imaging.

These findings align with previous research suggesting that US has a limited impact on patient management in cases of scrotal pain when physical examination is normal [2,4].

However, it's important to acknowledge the limitations of our study. As a retrospective analysis, it may be subject to selection bias. Additionally, the study was conducted at a single center, which may limit its generalizability to other settings with different levels of urological expertise.

## Conclusions

This study demonstrates the reliability of urological examinations in diagnosing testicular conditions, with reasonable positive predictive value. While ultrasonography remains a valuable tool, particularly in cases of suspected malignancy or equivocal clinical findings, our results suggest that routine use of US may not always be necessary when expert clinical assessment is available and is reassuring. We propose that when there is no clear clinical indication for a US scan, it may be more efficient to rely on expert clinical judgment and proper reassurance and support for the patient. However, in cases of scrotal lumps that have lasted for >14 days, we suggest GP practices pursue US imaging regardless of suspicion. This approach could potentially reduce unnecessary imaging, decrease healthcare costs, and improve the efficiency of urological services without compromising patient care.

Future prospective studies are needed to further validate these findings and to develop clear guidelines for the optimal use of ultrasonography in testicular assessments. Such research should aim to balance the need for diagnostic accuracy with resource utilization and patient convenience.
